# Incorporating periodic variability in hidden Markov models for animal movement

**DOI:** 10.1186/s40462-016-0093-6

**Published:** 2017-01-26

**Authors:** Michael Li, Benjamin M. Bolker

**Affiliations:** 1 Department of Biology, McMaster University, 1280 Main St. West, Hamilton, L8S 4K1 Ontario Canada; 2 Department of Mathematics and Statistics, McMaster University, 1280 Main St. West, Hamilton, L8S 4K1 Ontario Canada

**Keywords:** Hidden Markov model, Animal movement, Temporal autocorrelation, Temporal heterogeneity, Florida panther

## Abstract

**Background:**

Clustering time-series data into discrete groups can improve prediction and provide insight into the nature of underlying, unobservable states of the system. However, temporal variation in probabilities of group occupancy, or the rates at which individuals move between groups, can obscure such signals. We use finite mixture and hidden Markov models (HMMs), two standard clustering techniques, to model long-term hourly movement data from Florida panthers (*Puma concolor coryi*). Allowing for temporal heterogeneity in transition probabilities, a straightforward but little-used extension of the standard HMM framework, resolves some shortcomings of current models and clarifies the movement patterns of panthers.

**Results:**

Simulations and analyses of panther data showed that model misspecification (omitting important sources of variation) can lead to overfitting/overestimating the underlying number of movement states. Models incorporating temporal heterogeneity identify fewer underlying states, and can make out-of-sample predictions that capture observed diurnal and autocorrelated movement patterns exhibited by Florida panthers.

**Conclusion:**

Incorporating temporal heterogeneity improved goodness of fit and predictive capability as well as reducing the selected number of movement states closer to a biologically interpretable level, although there is further room for improvement here. Our results suggest that incorporating additional structure in statistical models of movement can allow more accurate assessment of appropriate model complexity.

**Electronic supplementary material:**

The online version of this article (doi:10.1186/s40462-016-0093-6) contains supplementary material, which is available to authorized users.

## Background

Given a sequence of animal movements, movement models aim to find a parsimonious description that can be used to understand past movements and predict future movements. Ecologists have long considered the effects of individual-level covariates (sex, age, nutritional status) and environmental covariates (habitat type, location of predators or prey) on movement [1–3]. More recently, modelers have developed *hidden Markov models* (HMMs) [4–6] — used in animal ecology under the rubric of the “multiphasic movement framework” [7] — that consider the effects of organisms’ *internal* states; in particular, HMMs model animal movement as though individual animals’ movement at particular times is determined by which of a discrete set of unobserved movement states (e.g. “foraging”, “traveling”, “resting”) they currently occupy. Conditional on the state occupied by an individual, HMMs typically assume that animals follow a correlated random walk model [8, 9].

Ever-increasing capabilities of remote sensors are making movement data available over an ever-wider range of time scales, at both higher resolution (e.g. hourly data from GPS collars vs. daily or weekly fixes for radio or VHF collars) and longer extent (e.g. from a few days to months or years). When analyzing such long-term data, ecologists will more often have to account for temporal variability in movement at diurnal and seasonal scales that were previously not captured in the data.

HMMs have typically been used to model movements over short time scales, where the probability of transitioning between movement states is approximately constant. Changes in transition probabilities based on the local environment can be accounted for by incorporating environmental covariates in the HMM [10], or inferred from direct comparisons between inferred states and environmental conditions [7]. Schliehe-Diecks et al. [11] considered temporal trends in behavioural transitions over the time scales of a six-hour observation period; for the most part ecologists have turned to other tools to describe behavioural changes over longer (diurnal, seasonal, or ontogenetic) time scales [12], although two recent papers have used HMMs with diurnal variation in transition probabilities to model shark behaviour [13, 14].

For animals that change their movement behaviour on a fast time scale, such that the steps between successive observations are effectively independent, *finite mixture models* (FMMs) — which can be considered a special case of HMMs where the probability of state occupancy is independent of the previous state — can adequately describe movement [15]. When movement varies over long time scales (relative to the time between observations) with little short-term persistence or correlation, movement could be well represented by FMMs where the occupancy probabilities change deterministically over time. Thus FMMs and HMMs, with or without temporal variation in the occupancy or transition probabilities, form a useful family of models for capturing changes in movement over a range of time scales.

Our primary goal in this paper is to discuss the use of HMMs with temporally varying transition probabilities — in particular, transition probabilities that follow a diurnal cycle — for modeling animal movement recorded over long time scales. In addition to simulation-based examples, we also re-analyze data from van de Kerk et al. [16], who used temporally homogeneous hidden semi-Markov models (HSMMs: an extension of HMMs that allow flexible modelling of the distribution of *dwell times*, the lengths of consecutive occupancy of a movement state) to describe the movement and putative underlying movement states of Florida panthers (*Puma concolor coryi*).

van de Kerk et al. [16] found that the best-fitting HSMMs incorporated a surprisingly large number of hidden movement states (as many as six for individuals with a large amount of available data); for reasons of computational practicality and biological interpretability, they restricted their detailed analysis to models with only three underlying states. In contrast, most studies using HMM have chosen the number of underlying states a priori, typically using either two [6, 7, 11, 17], or three states [18, 19]. (We know of two exceptions: Dean et al. [20] evaluated models with up to 10 states, but like van de Kerk et al. they chose to consider only models with three states. Langrock et al. [21] fitted models with up to 10 states; their goal, like ours, was to illustrate the potential for overfitting with misspecified models.) Behavioural repertoires with many distinct states are difficult to interpret — one reason that other authors have not adopted van de Kerk et al.’s model-based approach to identifying the number of movement states.

Our second goal, therefore, is to explore whether van de Kerk et al.’s results [16] on the number of movement states might be driven at least in part by shortcomings of their statistical model. For large data sets, the complexity penalties imposed by information-theoretic methods are often overwhelmed by the sheer amount of information (apparently) contained in the data, leading to selection of models with many parameters. HMMs typically use simple models for the behaviour within each movement state, e.g. two parameters each to describe the step-length and turning angle distributions. When the movement patterns in each state are more complex than the model allows for (e.g. animals behave differently as a function of spatial or temporal heterogeneity in the environment, or follow an unusual step-length distribution [21]), the model is forced to accommodate this complexity by subdividing animal movements into a large number of discrete movement states. We predict that increasing volumes of data will increasingly lead researchers who are accustomed to fitting small models to sparse data into such traps. We examine whether allowing for diurnal variation in the Florida panther data allows us to select models with fewer latent states; we also fit models to simulated data with varying numbers of latent states, and with and without temporal heterogeneity, to test our conjecture that heterogeneity can be misidentified as movement complexity. Finally, we discuss some of the conceptual and statistical difficulties underlying the general problem of estimating the number of discrete movement states underlying observed animal movement behaviour.

## Methods

### Data and previous analyses

GPS collars were fitted to 18 Florida panthers in 2005-2012 by Florida Fish and Wildlife and Conservation Commission staff using trained hounds and houndsmen. Of these animals, 13 had sufficient data to be used by van de Kerk et al. [16]. Here we focus on the four cats with the most data (all with approximately 10,000-15,000 observations: see Table 1), in part because our goal is to understand the issues that arise when simple models are fitted to large data sets, and in part because the general trend in telemetry studies is toward larger data sets. As is typical in studies of animal movement, we took first differences of the data by decomposing contiguous sequences of hourly GPS coordinates into successive step lengths (in meters) and turning angles (in radians) [9, 16]. As van de Kerk et al. did, we fitted a separate model to each individual cat trajectory; while mixed models would have more statistical power and would describe among-individual variation in a natural way [11], they do not improve estimates of individual movement parameters significantly in the case where a large amount of data is available for each individual [22].

**Table 1:** Cat ID and number of observations; ID numbers are given matching those shown by van de Kerk et al. [16] and those in the data located at the UF Institutional repository (IR@UF)

(van de Kerk 2015)	(IR@UF)	Number of observations
130	1	10286
131	2	9458
48	14	14645
94	15	10250

van de Kerk et al. [16] used hidden semi-Markov models (HSMM), an extension of HMM that permits explicit modelling of dwell times [6], considering both Poisson and negative binomial distributions for dwell times. As shown by van de Kerk et al. [16], the estimated shape parameter of the negative binomial dwell time distribution was typically close to 1 (≈0.4−1.6; confidence intervals were not given), implying that a geometric distribution (i.e., negative binomial with shape=1) might be adequate. For computational simplicity, therefore (the computational tools we use below do not easily allow for HSMMs) we reverted to the simpler HMM framework which assumes fixed switching rates, and hence geometrically distributed dwell times.

van de Kerk et al. [16] considered time-homogeneous models with a variety of candidate distributions — log-Normal, Gamma, and Weibull distributions for step lengths and von Mises and wrapped Cauchy distributions for the turning angle — concluding on the basis of the Akaike information criterion (AIC) that Weibull step length and wrapped Cauchy turning angle distributions were best. Since our analysis aims for simplicity and qualitative conclusions rather than for picking the very best predictive model, we focus on models that treat each step as a univariate, log-Normally distributed observation, glossing over both the differences in shape between the three candidate step-length distributions and the effects of considering multivariate (i.e., step length plus turning angle) observations. To check that this simplification does not distort our conclusions we do briefly compare log-Normal and Weibull step-length distributions, with and without a von Mises-distributed turning angle included in the model (Fig. 2); we also repeated the analysis with turning angles included for most models (Additional file 1).

van de Kerk et al. [16] used the Bayesian (Schwarz) information criterion (BIC) to test the relative penalized goodness of fit for models ranging from 2 to 6 latent states. In general, BIC values decreased as the number of states increased from three to six states, suggesting that the six-state model was favoured statistically; however, the authors used three-state models in most of their analyses for ease of biological interpretation. We follow van de Kerk et al. [16] in using BIC-optimality (i.e., minimum BIC across a family of models) as the criterion for identifying the best model, because we are interested in explaining the data generation process by identifying the “true” number of underlying movement states.

Using BIC also simplifies evaluation of model selection procedures; it is easier to test whether our model selection procedure has selected the model used to simulate the data, rather than testing whether it has selected the model with the minimal Kullback-Leibler distance [23]. We recognize that ecologists will often be interested in maximizing predictive accuracy rather than selecting a true model, and that as usual in ecological systems the true model will be far more complicated than any candidate model [24]. We have repeated some of our analyses using AIC rather than BIC (not shown); for our examples, the qualitative conclusions stated here for BIC-optimality carry over to analyses using AIC.

### Model description

In a HMM, the joint likelihood of *emissions* (i.e., direct observations) **Y**=**y**_1_,…,**y**_*T*_ and a hidden state sequence **Z**,*z*_*t*_∈{1,…,*n*},*t*=1,…,*T*, given model parameters ***θ*** and covariates **X**_1:*T*_=**x**_1_,…,**x**_*T*_, can be written as:

1 $$ \begin{aligned} P \left(\mathbf{Y}_{1:T},\mathbf{Z}_{1:T}|\boldsymbol{\theta},\mathbf{X}_{1:T} \right) & = P(z_{1} \mid \mathbf{x}_{1}) P \left(\mathbf{y}_{1} | z_{1}, \mathbf{x}_{1}\right) \\ & \quad \times \prod\limits_{k=2}^{T} P\left(z_{k} | z_{k-1}, \mathbf{x}_{k}\right) P\left(\mathbf{y}_{k} | z_{k},\mathbf{x}_{k}\right) \end{aligned}  $$

The emissions **y**_*i*_ are boldfaced to denote that we may have a vector of observations at each time point (e.g., step length and turning angle). The model contains three distinct components:

Initial probability *P*(*z*_1_=*i*|**x**_1_)*P*(**y**_1_|*z*_1_,**x**_1_): the probability of state *i* at time *t*=1 given that the covariates are **x**_1_, times the vector of observations **y**_1_ conditioned on state *z*_1_ and covariates **x**_1_.
Transition probability *P*(*z*_*k*_=*j*|*z*_*k*−1_=*i*,**x**_*k*_): the probability of a transition from state *i* at time *t*=*k*−1 to state *j* at time *t*=*k*, given covariates **x**_*k*_.
Emission probability *P*(**y**_*k*_|*z*_*k*_,**x**_*k*_): a vector of observations **y**_*k*_ given state *z*_*k*_ at time *t* = *k* and covariates **x**_*k*_.

Equation 1 gives the likelihood of the observed sequence given (conditional on) a particular hidden sequence. In order to calculate the overall, unconditional (or marginal) likelihood of the observed sequence, we need to average over all possible hidden sequences. There are several efficient algorithms for computing the marginal likelihood and numerically estimating parameters [25]; we used those implemented in the depmixS4 package for R [26, 27].

For an *n*-state HMM, we need to define an *n*×*n* matrix that specifies the probabilities *π*_*ij*_ of being in movement states *j* at time *t*+1 given that the individual is in state *i* at time *t*. The FMM is a special case of HMM where the probabilities of *entering* a given state are identical across all states — i.e., the probability of occupying a state at the next time step is independent of the current state occupancy. It can be modelled in the HMM framework by setting the transition probabilities *π*_*ij*_=*π*_*i*∗_ for *j*=1,…,*n*.

In any case, the transition matrix *π*_*ij*_ must respect the constraints that (1) all probabilities are between 0 and 1 and (2) transition probabilities out of a given state sum to 1. As is standard for HMMs with covariates [26], we define this multinomial logistic model in terms of a linear predictor *η*_*ij*_, where *η*_*i*1_ is set to 1 (i.e. we have only *n*×(*n*−1) distinct parameters; we index *j* from 2 to *n* for notational clarity):

2 $$  \begin{aligned} \pi_{ij} & = \exp(\eta_{ij}(t))/\left(1+\sum\limits_{j=2}^{n}\exp(\eta_{ij}(t))\right), \mathrm{for~} j={2,\ldots,n} \\ \pi_{i1} & = 1 - \sum\limits_{j=2}^{n}\pi_{ij} \end{aligned}  $$

We considered four different transition models for diurnal variation in movement, incorporating hour-of-day as a covariate following the general approach of Morales et al. [18] of incorporating covariate dependence in the transition matrix.

Multiple block transition Here we assume piecewise-constant transition probabilities. The transition probability *π*_*ij*_ is a function of time (hour of day), where it is assigned to one of *M* different time blocks:
  3 $$\begin{array}{*{20}l} \eta_{ij}(t) = \sum\limits_{m=1}^{M}a_{ijm} \delta_{m=t} \end{array} $$
  where *a*_*ijm*_ are parameters, and *δ*_*m*=*t*_ is a Kronecker delta (*δ*_*m*=*t*_=1 for the time block corresponding to time *t*, and 0 otherwise).
Quadratic transition model We assume the elements of the linear predictor are quadratic functions of hour:
  4 $$\begin{array}{*{20}l} \eta_{ij}(t) = b_{ij1}+b_{ij2}\left(\frac{t}{24}\right)+b_{ij3}\left(\frac{t}{24}\right)^{2}. \end{array} $$
  The quadratic model is not diurnally continuous, i.e. there is no constraint that forces *η*_*ij*_(0)=*η*_*ij*_(24); imposing a diurnal continuity constraint would collapse the model to a constant.
Sinusoidal transition model A sinusoidal model with a period of 24 hours is identical in complexity to the quadratic model, but automatically satisfies the diurnal continuity constraint:
  5 $$\begin{array}{*{20}l} \eta_{ij}(t)= b_{ij1}+b_{ij2}\cos\left(\frac{2\pi t}{24}\right)+b_{ij3}\sin\left(\frac{2\pi t}{24}\right). \end{array} $$
  Similar models have been used in recent HMM studies of shark behaviour [13, 14].
Hourly model Lastly, we extended the multi-block approach and assigned a different transition matrix for every hour of the day (i.e. this is a special case of the multi-block model with *M*=24). This model is included for comparative purposes; due to the large number of parameters in the model (more than 24*n*(*n*−1) for a HMM with *n* states), it is not really practical. We only fitted up to four states using the hourly model.

Other periodic functions, such as Fourier series (i.e., the sinusoidal transition model augmented by additional sinusoidal components at higher frequencies) or periodic splines, could also be considered.

Model complexity and the number of parameters increase as the number of latent states increase. For a fixed number of states homogeneous FMMs are simplest, followed by homogeneous HMMs and finally by FMMs and HMMs incorporating temporal heterogeneity. In general, the number of free parameters in an HMM is the sum of the number of free parameters for each of the three model components (initial states, transition probabilities, and emissions). Let *n* be the number of hidden states and *k*_*i*_,*k*_*t*_,*k*_*e*_ be the number of parameters describing the covariate-dependence of the prior distribution, transition function and emission distributions; that is, for a homogeneous model, *k*=1, while a single numeric covariate or a categorical predictor with two levels would give *k*=2. Then the number of free parameters of an HMM is: [*Initial states*] *k*_*i*_·(*n*−1) + [*Transition probabilities*] *k*_*t*_·*n*·(*n*−1) + [*Emission parameters*] *k*_*e*_·*n*. As the number of states increases, the number of free parameters in (homogeneous or heterogeneous) FMMs and time-homogeneous HMMs increases linearly, whereas for HMMs with temporal heterogeneity (or covariate-dependent transitions more generally) the number increases quadratically.

For most of our analyses, we followed van de Kerk et al. in assuming that GPS error was negligible, i.e. that step lengths and turning angles could be measured without error. However, we did one set of simulations to check the effect of GPS error on our conclusions. In this case, we reconstructed the spatial coordinates simulated from the models above: if the step length and turning angles chosen from one of the models above are denoted as {*s*_*t*_,*σ*_*t*_} then

6 $$ \begin{aligned} x_{t+1} & = x_{t} + s_{t} \cos \sigma_{t} \\ y_{t+1} & = y_{t} + s_{t} \sin \sigma_{t}. \end{aligned}  $$

We then added radially symmetric GPS noise (*x*_*t*,obs_=*x*_*t*_+*N*(0,5), *y*_*t*,obs_=*y*_*t*_+*N*(0,5)) and reconstructed the observed step-length sequences from the sequence of noisy positions (See Additional file 2 for more details).

Incorporating both hidden behavioural states (with temporally heterogeneous transitions) and GPS error in the same statistical model would require combining the standard discrete HMM framework with an additional hierarchical layer describing the true the {*x,y*} coordinates as a continuous, bivariate latent variable. While this would probably be possible using a Bayesian MCMC method, it would be challenging and seems to be rarely attempted [28, 29]. We instead treat this case as a robustness analysis, fitting the noisy simulation with a model that ignores the noise to assess the effects of noise on our conclusions [30].

### Model evaluation

We used the depmixS4 package [26] to fit covariate-dependent transition HMMs, simulate states and step lengths using the estimated parameters, and estimate the most likely sequence of movement states with the Viterbi algorithm.

We ran a simulation experiment in which we fitted HMMs with both homogeneous and heterogeneous transition probabilities to simulated data with both cases to see whether the correct (heterogeneous-transition) model correctly identified the number of states while the misspecified (homogeneous-transition) model overestimated the number of states. We also included two additional experiments with additional errors. We used 100 realizations of the four cases, fitting each realization with HMMs ranging from 2 to 4 movement states, with and without temporal heterogeneity in the transition probabilities.

We used three approaches to assess the fit of both time-homogeneous and time-inhomogeneous HMMs with 3 to 6 states to step-length data from the four of the thirteen Florida panthers with the most data (>9000 observations). (1) BIC was used to compare the goodness of fit of each model type. The model with the lowest BIC was selected to be the optimal-BIC model and all BICs were adjusted to *Δ*BIC based on the optimal-BIC model (*Δ*BIC = BIC - min(BIC)). (2) Comparing average step-length by hour of day for the observed data and for data simulated from the models shows how well a particular class of models can capture diurnal variation in movement. (3) Comparing temporal autocorrelations for the observed data and for data simulated from the models shows how well a particular class of models can capture serial correlation at both short and long time scales. For multiple block transition HMMs, we selected three blocks (*M*=3) based on the similarities of average movement by time of day within each block (*m*_1_=21−23,0−6,*m*_2_=7−16,*m*_3_=17−20). As a complement, we also fitted FMM and FMM with priors on state occupancy that varied sinusoidally over time to compare the temporal effects in goodness of fit. As a reminder, FMMs assume that the latent state in each time step is *independent* of the latent state at the previous time step; time-varying FMMs can accurately describe movement change on a short time scale, but the average propensity for different movement changes over time.

While the expected step length and ACF can be computed directly from estimated parameters for FMMs and (with some difficulty) homogeneous HMMs, the interaction between the geometric dwell time within each state and the temporally varying interaction probabilities makes this calculation infeasible for more complex models. Therefore, we used simulations to predict expected hourly step lengths and autocorrelation functions (ACF). Specifically, we first simulated the movement states forward by choosing a multinomial sample for each time step, using the estimated model parameters and conditioning on the previous movement state and (for heterogeneous models) on the time of day. We then sampled step lengths independently for each step, conditioning on the simulated movement state. Finally, we computed average step lengths and ACFs from the realizations, which were run for the same number of steps as the corresponding data set (long enough to make the starting conditions negligible). We compared our simulated predictions with the observed movements. For comparison, we also simulated step lengths based on the Viterbi estimates of the states occupied by time of day in the observed data. This approach of generating predictions from the expected or simulated step lengths/turning angles conditional on the most likely state sequence predicted by the Viterbi algorithms, or using predictions based on pseudo-residuals [6, 25], is problematic in some applications because the predictions condition on the observed data. While it is useful for predicting missing data in the observation sequence, it may not be reliable for evaluating the goodness of fit for HMM models with different degrees of structural complexity.

## Results

**Fig. 1:**
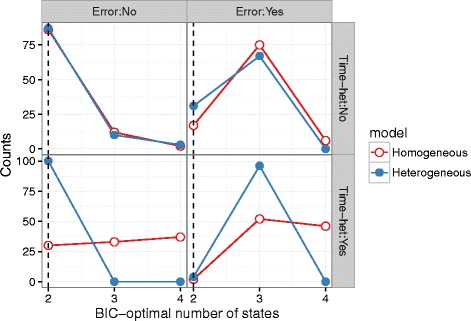
Frequency of BIC-optimal numbers of states estimated, from 100 realizations of step lengths from an HMM with two latent states. In general misspecification increases the frequency of overfitting (i.e. selecting more states than the true value of *n*=2), whether temporal heterogeneity is neglected (*red lines*, *bottom row*) or observation error is not accounted for (*right column*). Fitting models may incorporate temporal heterogeneity in transition probabilities (*blue lines*) or exclude it (*red lines*). Subplots show variation in (*left*/*right*) whether error is added to the observed step lengths and (*top*/*bottom*) whether the simulation model incorporates temporal heterogeneity

The simulation experiment supports our hypothesis that homogeneous transition HMMs can overestimate the number of hidden states when the model is misspecified (Fig. 1 bottom left panel) without GPS error. Heterogeneous transition models can always predict the correct number of states (in 100/100 simulations, BIC correctly identifies *n*=2 as the number of states), whereas the temporally homogeneous models overestimate the number of states (the correct value, *n*=2, is chosen most often, but in fewer than half of the simulations; values up to *n*=5 are frequently chosen).

GPS noise has similar effects to temporal heterogeneity. When GPS noise is added to temporally homogeneous simulations with *n*=2, both model classes (with and without temporal heterogeneity in transition probabilities) choose *n*=3 about 75% of the time. In simulations with temporal heterogeneity, temporally heterogeneous models choose *n*=3 in nearly all cases, while homogeneous models choose *n*=3 and *n*=4 equallty.

**Fig. 2:**
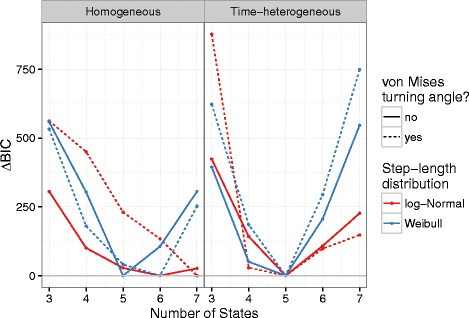
Comparison of BIC-optimal state predictions for panther data for homogeneous transition HMMs (*left panel*) and heterogeneous transition HMMs (*right panel*), with movement step distribution models of varying complexity. For the time-heterogeneous model, all model variants estimate the same BIC-optimal number of behavioural modes (5); the homogeneous model is more variable, with the number of modes ranging from 5 to 7 or higher. *Solid line*: univariate response HMMs (turning angles ignored); *dotted line:* multivariate response HMMs (turning angles included, assuming a von Mises distribution). *Red lines*: log-normal step-length distribution; *blue lines*: Weibull step-length distribution

In the real data application, the BIC-optimal number of states for time-homogeneous models is consistent with van de Kerk et al.’s [16] results. For time-homogeneous models, the Weibull-wrapped-Cauchy [16], Weibull-von Mises, and log Normal without turning angles all identify the same BIC-optimal number of states. While the number of states identified by homogeneous-HMM models varies according to the step-length/turning-angle distributions chosen, ranging from *n*=5 for Weibull steps alone to *n*=7 for the log Normal-von Mises emissions model, the number of states identified by heterogeneous-HMM models is consistent among step-length/turning-angle models (*n*=5: Fig. 2). In general the models with turning angles select slightly higher BIC-optimal numbers of states because they have more data to work with (two observations per time step rather than one), hence the relative importance of the goodness-of-fit (likelihood) increases relative to the complexity penalty. Cats 2, 14, and 15 show similar patterns, although there is more variation, especially among step-length models; in general the heterogeneous models are more consistent than the homogeneous models (see Additional file 3). (Across the board, all four cats analyzed showed similar results, so we present results for Cat 1 only throughout. Analogous results for the other three cats are available in the Additional file 3.)

**Fig. 3:**
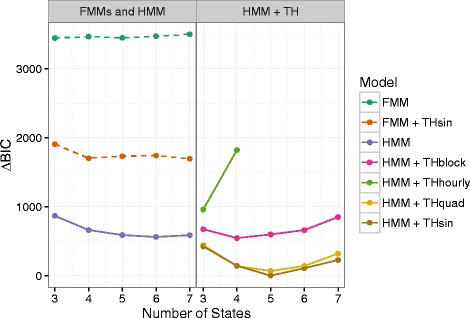
Adjusted BIC for all numbers of states. With the exception of the hourly model, all temporally heterogeneous HMMs give better/more parsimonious fits to the data, as well as selecting BIC-optimal models with fewer states. *Left panel*: homogeneous FMM, heterogeneous FMM (sinusoidal prior), and homogeneous HMM. *Right panel*: HMMs with different temporal transition models. *Dashed lines*: FMMs; *solid lines*: HMMs

Models with temporal heterogeneity provide better fits to the data (lower BIC) than homogeneous models in both FMM and HMM frameworks, but time-homogeneous HMMs are better than FMMs with sinusoidal temporal heterogeneity (Fig. 3). Turning to the temporally heterogeneous HMMs (Fig. 3, right panel), we see that the model with different transition probabilities for each hour of the day (HMM + THhourly) is overparameterized; it underperforms homogeneous HMM with even 3 states, and gets much worse with 4 states. The multiple-block model gives approximately the same BIC as the homogeneous HMM, although it gives the BIC-optimal number of states as 4, in contrast to 6 for the homogeneous HMM. Finally, the quadratic and sinusoidal models are the best models tested by far; they both give the BIC-optimal number of states as 5, and they have similar goodness of fit. However, the similarity between the quadratic and sinusoidal models may be overstated in Fig. 3 due to the very large variation in BIC (over thousands of units) across the full range of models; the best-fit sinusoidal (*n*=5) model is approximately 80 BIC units better than the best quadratic model (also *n*=5), which would normally be interpreted as an enormous improvement in goodness of fit (both models have 90 parameters).

These conclusions persist across the other cats, and when turning angles are included in the model (Additional file 1). Temporally heterogeneous models select fewer BIC-optimal states (4 or 5) than homogeneous models (6 or ≥7). The best overall model is usually the sinusoidal heterogeneous model. The only exception is for the cats with the least data, where the greater complexity of the heterogeneous models has a stronger effect; the homogeneous model wins overall for cat #15 (step-length-only models) and for cats #14 and #15 (models with step length and turning angle).

**Fig. 4:**
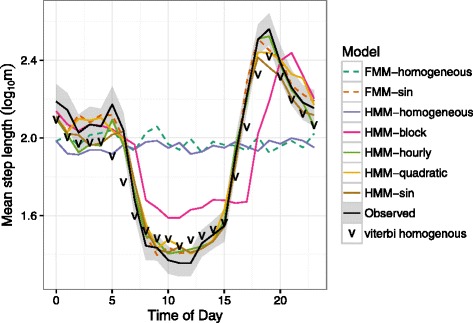
Average step-length by time of day from out-of-sample predictions for BIC-optimal models in each category. All of the temporally heterogeneous models with the exception of the block model fit the observed pattern adequately, as does the prediction based on Viterbi residuals (which conditions on the observed step lengths, so is not truly out-of-sample); the temporally homogeneous models fail for all four cats. *Dashed lines*: FMMs; *solid lines*: HMMs; v *points*: within-sample Viterbi predictions of a three-state homogeneous HMM. *Black line* and *grey ribbon* show observed average step length by hour, ± two SE

The average hourly step lengths from the observed panther data exhibit a clear diurnal pattern (Fig. 4). As expected, temporally homogeneous models (whether FMM or HMM) predict the same mean step length regardless of time of day, failing to capture the diurnal activity cycle. All of the models incorporating temporal heterogeneity, including the temporally heterogeneous FMM, can capture the observed patterns. However, the block model does markedly worse than the other temporal models (changing the block definitions might help by re-clustering/grouping different hours or increasing the number of blocks), and the (overparameterized) hourly model does better than any other model at capturing the early-evening peak (but worse at capturing the mid-day trough). We also included average hourly step lengths from three-state temporally homogeneous HMM Viterbi prediction to illustrate within sample predictions can capture the diurnal patterns, but fail to capture out of sample predictions.

**Fig. 5:**
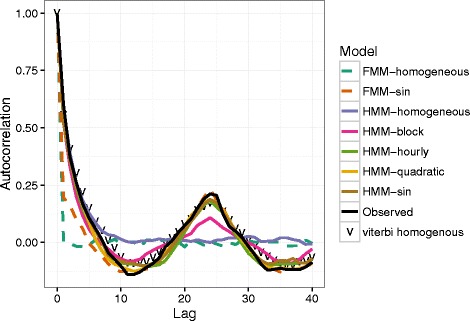
Out-of-sample predicted autocorrelations for BIC-optimal models in each category. All of the HMMs, whether temporally homogeneous or heterogenous, fit the short-term (lag <10 hour) pattern adequately: all of the temporally heterogeneous models, whether FMM or HMM, fit the long-term (lag >10 hour) pattern adequately. *Dashed lines*: FMMs; *solid lines*: HMMs; v *points*: within-sample Viterbi predictions of a three-state homogeneous HMM. *Black line* shows observed average step length by hour

Like the diurnal pattern (Fig. 4), the strong autocorrelation of the observed step lengths at a 24-hour lag (Fig. 5) shows the need to incorporate temporal heterogeneity in the model — we could have reached this conclusion even without developing any of the temporal-heterogeneity machinery. In contrast to the hourly averages, the autocorrelation (ACF) captures both short- and long-term temporal effects. HMM without temporal heterogeneity captures the short-term autocorrelation, but misses the long-term autocorrelation beyond a 7-hour lag. Temporally homogeneous FMMs, by definition, produce no autocorrelation (neither short- nor long-term autocorrelation). FMMs without temporal heterogeneity, although they capture the diurnal pattern well, underpredict the degree of short-term autocorrelation.

**Fig. 6:**
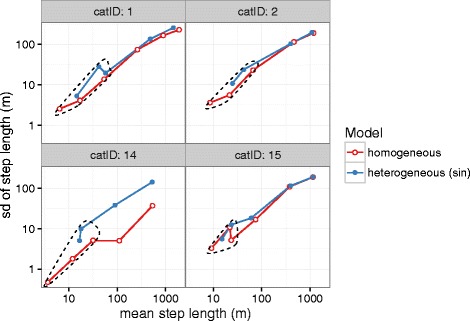
Comparison of step length distribution parameters for BIC-optimal HMMs (homogeneous and sinusoidal) for four panthers. Homogeneous and heterogeneous models agree qualitatively on long-step-length state parameters; homogeneous models subdivide short-step-length states into an additional category. Mean (*x*-axis) and standard deviation (*y*-axis) of step length by state (log-Normal parameters, units of log10m) for BIC-optimal homogeneous HMMs (*red line*) and heterogeneous HMMs with sinusoidal transition (*blue line*). *Dashed circles* highlight comparable sets of short-step-length movement states

Perhaps the hardest part of analysis is drawing reasonable biological conclusions about the movement states identified by the model. Where we previously confined our attention to the single cat with the most data, we now compare the estimated emission parameter values (mean and standard deviation of the step length in each state) between the homogeneous and heterogenous models for all four cats in our sample (Fig. 6). Our goal is to understand why the homogeneous models have identified more states than the heterogeneous models: how are the heterogeneous models able to group behaviours that the homogeneous models have separated into different modes? In general, the states with longer mean step lengths are similar between homogeneous and heterogeneous models. For cats 14 and 15, the states with the longest or next-longest mean step lengths have similar means and standard deviations; for cats 1 and 2, three long-step states in the homogeneous HMM appear to divide two long-step states in the heterogeneous HMM. For short-step states, the heterogeneous HMM tends to identify a high-variance state, while the homogeneous HMM picks up states with very short step lengths. That is, the homogeneous HMM analysis has concluded (at least for cats 2, 14, and 15) that a cluster of short-distance moves can be subdivided into three separate states, while the heterogeneous HMM analysis identifies only two sub-states. We believe this occurs because of additional residual autocorrelation in the homogeneous HMM, which would lead to an inflation of the difference in the model log-likelihoods and thus lead the models to pick more complex models. Finally, we suspect that some additional overspecification of the short-range movemement behaviour may be due to GPS error [30], which is not specifically accounted for in our model.

## Discussion

HMMs are a widely used and flexible tool for modeling animal movement; we need to work harder to make sure they are both appropriately complex and biologically interpretable. With the increasing volumes of movement data available, ecologists who naively use traditional homogeneous HMMs and standard information-theoretic criteria to estimate the number of movement states will generally overfit their data, i.e. they will “discover” large number of states that are difficult to interpret biologically. Our results agree with those of Langrock et al. [21], who consider the effects of misspecification in the step-length distribution, i.e. of inappropriately assuming the step lengths in each state follow some particular parametric distribution.

As usual, the appropriate approach depends on the goal of the analysis. If ecologists simply want to identify states and associate them with environmental characteristics, it might be sufficient to use a simple (homogeneous) H(S)MM model, pre-specifying the number of states to a biologically sensible value, and then match *post hoc* Viterbi estimates of state occupancy with environmental variation in space and time [7]. For example, the conclusion that panthers are more likely to move long distances at night (as well as being long known to panther biologists) was reached by van de Kerk et al. by averaging Viterbi estimates of state occupancy by time of day ([16], Fig. 4). On the other hand, if the goal of analysis is to make out-of-sample predictions about animal movement, such as in a management context, it is necessary to fit a covariate-dependent model that explicitly incorporates the switching process. While the Viterbi algorithm can be applied to work backward from observed movement to variations in state occupancy with environmental conditions even when using a homogeneous model, a homogeneous model can never *predict* movement that varies with environmental conditions.

If our goal is actually to estimate how many different kinds of movement are within a given species’ behavioural repertoire — keeping in mind that these discrete movement states are certainly an oversimplified representation of animals’ real internal states — then, as we have shown above, relatively complex models will generally be required to avoid overestimating the number of states. More generally, we question whether estimating the number of discrete modes, rather than deciding on the number of states a priori, is a sensible procedure. With enough data, animal behaviour is probably subdivisible into arbitrarily many discrete modes. This recalls Burnham and Anderson’s “tapering effects” concept [31]: the “true” model for most ecological systems is effectively infinite-dimensional. While adding covariates can help explain some behavioural variation and reduce the number of estimated modes, it does not address the underlying tapering-effects problem. Information-theoretic analysis of the number of modes could be useful for purely predictive models, or possibly — if the analysis gave strong support for more discrete, biologically interpretable behavioural categories than were previously identified from direct natural-history observations — to identify cryptic behavioural modes. In attempting to assign biological meaning to movement modes identified by models, we have graphically explored the parameter values (e.g. Fig. 6) as well as the spatial and temporal distributions of Viterbi-estimated state occupancy; our failure to see any clear patterns in these analyses contributed to our previous decisions to revert to the a priori guess of three movement states. In any case, researchers should not take optimal numbers of modes identified by such analysis at face value, especially for large data sets.

On a more technical note, researchers in cluster analysis (of which HMMs are a special case) have shown that the technical conditions required for BIC to apply may be violated [32] — because a model can contain a movement state that is never used, leading to transition probabilities of zero that correspond to parameters on the boundary of the allowable parameter space. However, BIC can be useful as an approximate *upper* limit on the number of states. Various solutions to this problem have been proposed, including the “integrated classification likelihood” (ICL) [32, 33], as well as a simpler “knee point” method [34] that looks for the cluster size that corresponds to the largest change in BIC rather than to the smallest overall BIC. (Dean et al. [20] took a similar approach, but based on the log-likelihood curve rather than the BIC.) Nevertheless, in our simulations the BIC does correctly identify the number of states when appropriate heterogeneity is included in the model.

Animal movement models are becoming more complex, and the list of processes that can be incorporated in these models is almost unlimited. We focused on the effects of temporal heterogeneity because it was an obvious driver of behaviour that had been omitted from previous analyses. We neglected several other covariates that could clearly affect panther movement (e.g. local habitat type and distance from roads; distance from home range center; presence of nearby conspecifics; previous occupancy history). We partly or completely neglected other phenomena such as the information provided by turning angles; GPS error [35]; and non-geometric dwell times (HSMMs). Omission of any of these processes means that our models are misspecified — animals can behave in ways that are not captured by the model — and thus subject to overestimating the number of latent states.

Our models incorporating temporal heterogeneity identify more BIC-optimal states than we can easily assign biological meanings to, presumably due to additional sources of complexity that we did not include in our model. With sufficient data, computational resources, and programming resources, we could in principle have included many more processes in our model. However, data requirements, computation time and numerical instability of complex models, and the complexity of model selection and interpretation all make this approach impractical in reality [36]. Biologists should instead aim to prioritize the processes they incorporate in movement models based on their natural history knowledge, using graphical and quantitative diagnostics to test for robustness of the fit. Better diagnostic procedures and tests are needed: although it is important to assess overall goodness-of-fit [37], it is even more important to localize fitting problems to particular aspects of the data so that models can be constructed without needing to include all possible features of interest. Pseudo-residuals can be useful for this purpose [6], but researchers should note that they condition on the observed step lengths and turning angles and hence can be optimistic in some cases; posterior predictive simulations, which compare distributions of summary statistics from model simulations to observed values, may be a useful alternative [38].

## Conclusion

We have presented a simple but little-used extension (time-dependent transitions) to HMMs that partly resolves problems of overfitting the number of discrete movement states that underly the movements of Florida panthers. Time-dependent transitions offer a simple way to (1) reduce the selected number of states closer to a biologically interpretable level; (2) capture observed diurnal and autocorrelation patterns in a model that can make out-of-sample predictions; (3) improve overall model fit (i.e., lower BIC) and reduce the level of complexity (number of parameters) of the most parsimonious models. More generally, we have added an option to the expanding menu of modeling options available to movement ecologists within the hidden Markov model framework. By choosing thoughtfully from this menu, ecologists will better be able to quantify the behaviour of their focal species.

## Additional files

Additional file 1 Log-Normal Von Mises HMMs. (PDF 231 kb)

Additional file 2 Simulation experiment. (PDF 58.8 kb)

Additional file 3 HMM Analysis for Cat 2, 14, 15. (PDF 90 kb)

## References

[1] Patterson TA, Thomas L, Wilcox C, Ovaskainen O, Matthiopoulos J (2008). State–space models of individual animal movement. Trends Ecol Evol.

[2] McKenzie HW, Lewis MA, Merrill EH (2009). First passage time analysis of animal movement and insights into the functional response. Bull Math Biol.

[3] Pal S, Ghosh B, Roy S (1998). Dispersal behaviour of free-ranging dogs (*Canis familiaris*) in relation to age, sex, season and dispersal distance. Appl Anim Behav Sci.

[4] Firle S, Bommarco R, Ekbom B, Natiello M (1998). The influence of movement and resting behavior on the range of three carabid beetles. Ecology.

[5] Nathan R, Getz WM, Revilla E, Holyoak M, Kadmon R, Saltz D, Smouse PE (2008). A movement ecology paradigm for unifying organismal movement research. Proc Natl Acad Sci.

[6] Langrock R, King R, Matthiopoulos J, Thomas L, Fortin D, Morales JM (2012). Flexible and practical modeling of animal telemetry data: hidden Markov models and extensions. Ecology.

[7] Fryxell JM, Hazell M, Börger L, Dalziel BD, Haydon DT, Morales JM, McIntosh T, Rosatte RC (2008). Multiple movement modes by large herbivores at multiple spatiotemporal scales. Proc Natl Acad Sci.

[8] Okubo A, Smon AL. Diffusion and ecological problems: modern perspectives. Vol. 14: Springer Science & Business Media; 2013.

[9] Turchin P (1998). Quantitative Analysis of Movement: Measuring and Modeling Population Redistribution in Animals and Plants.

[10] Patterson TA, Basson M, Bravington MV, Gunn JS (2009). Classifying movement behaviour in relation to environmental conditions using hidden Markov models. J Anim Ecol.

[11] Schliehe-Diecks S, Kappeler PM, Langrock R (2012). On the application of mixed hidden Markov models to multiple behavioural time series. Interface Focus.

[12] Gurarie E, Andrews RD, Laidre KL (2009). A novel method for identifying behavioural changes in animal movement data. Ecol Lett.

[13] Towner AV, Leos-Barajas V, Langrock R, Schick RS, Smale MJ, Kaschke T, Jewell OJD, Papastamatiou YP (2016). Sex-specific and individual preferences for hunting strategies in white sharks. Funct Ecol.

[14] Leos-Barajas V, Photopoulou T, Langrock R, Patterson TA, Watanabe YY, Murgatroyd M, Papastamatiou YP. Analysis of animal accelerometer data using hidden Markov models. Methods Ecol Evol. 2016. doi:10.1111/2041-210X.12657. Accessed 23 Dec 2016.

[15] Tracey JA, Zhu J, Boydston E, Lyren L, Fisher RN, Crooks KR (2012). Mapping behavioral landscapes for animal movement: a finite mixture modeling approach. Ecol Appl.

[16] van de Kerk M, Onorato DP, Criffield MA, Bolker BM, Augustine BC, McKinley SA, Oli MK (2015). Hidden semi-Markov models reveal multiphasic movement of the endangered Florida panther. J Anim Ecol.

[17] McKellar AE, Langrock R, Walters JR, Kesler DC. Using mixed hidden Markov models to examine behavioral states in a cooperatively breeding bird. Behav Ecol. 2014; 171. doi:10.1093/beheco/aru171.

[18] Morales JM, Haydon DT, Frair J, Holsinger KE, Fryxell JM (2004). Extracting more out of relocation data: building movement models as mixtures of random walks. Ecology.

[19] Franke A, Caelli T, Kuzyk G, Hudson RJ (2006). Prediction of wolf (*Canis lupus*) kill-sites using hidden Markov models. Ecol Model.

[20] Dean B, Freeman R, Kirk H, Leonard K, Phillips RA, Perrins CM, Guilford T. Behavioural mapping of a pelagic seabird: combining multiple sensors and a hidden Markov model reveals the distribution of at-sea behaviour. J R Soc Interface. 2012. doi:10.1098/rsif.2012.0570. 10.1098/rsif.2012.0570 PMC3565783 23034356

[21] Langrock R, Kneib T, Sohn A, DeRuiter SL (2015). Nonparametric inference in hidden Markov models using P-splines: nonparametric inference in Hidden Markov Models. Biometrics.

[22] Bolker BM. Ecological Statistics: Contemporary Theory and Application In: Fox GA, Negrete-Yankelevich S, Sosa VJ, editors. Oxford: Oxford University Press: 2015. p. 310–34.

[23] Richards SA (2005). Testing ecological theory using the information-theoretic approach: examples and cautionary results. Ecology.

[24] Burnham KP, Anderson DR (1998). model selection and inference: a practical information-theoretic approach.

[25] Zucchini W, MacDonald IL (2009). Hidden Markov Models for Time Series: An Introduction Using R.

[26] Visser I, Speekenbrink M (2010). depmixS4: An R package for hidden Markov models. J Stat Softw.

[27] R Core Team (2015). R: A Language and Environment for Statistical Computing.

[28] Pedersen MW, Righton D, Thygesen UH, Andersen KH, Madsen H (2008). Geolocation of North Sea cod (*Gadus morhua*) using hidden Markov models and behavioural switching. Can J Fish Aquat Sci.

[29] Jonsen ID, Basson M, Bestley S, Bravington MV, Patterson TA, Pedersen MW, Thomson R, Thygesen UH, Wotherspoon SJ (2013). State-space models for bio-loggers: A methodological road map. Deep Sea Res Part II: Topical Stud Oceanogr.

[30] Bradshaw CJ, Sims DW, Hays GC (2007). Measurement error causes scale-dependent threshold erosion of biological signals in animal movement data. Ecol Appl.

[31] Burnham KP, Anderson DR (1998). Model selection and inference: a practical information-theoretic approach.

[32] Biernacki C, Celeux G, Govaert G (2000). Assessing a mixture model for clustering with the integrated completed likelihood. IEEE Trans Pattern Anal Mach Intell.

[33] Celeux G, Durand JB (2008). Selecting hidden Markov model state number with cross-validated likelihood. Comput Stat.

[34] Zhao Q, Xu M, Fränti P. Knee Point Detection on Bayesian Information Criterion. In: 2008 20th IEEE International Conference on Tools with Artificial Intelligence. IEEE: 2008. p. 431–8. doi:10.1109/ICTAI.2008.154, http://ieeexplore.ieee.org/lpdocs/epic03/wrapper.htm?arnumber=4669805.

[35] Hurford A (2009). GPS measurement error gives rise to spurious 180 ° turning angles and strong directional biases in animal movement data. PLOS ONE.

[36] Patterson TA, Parton A, Langrock R, Blackwell PG, Thomas L, King R. Statistical modelling of animal movement: a myopic review and a discussion of good practice. 2016. arXiv:1603.07511 [q-bio, stat]. Accessed 20 Dec 2016.

[37] Potts JR, Auger-Méthé M, Mokross K, Lewis MA (2014). A generalized residual technique for analysing complex movement models using earth mover’s distance. Methods Ecol Evol.

[38] Kramer M (2014). Use of the posterior predictive distribution as a diagnostic tool for mixed models.

