# Supplementary material for: Incorporating periodic variability in hidden Markov models for animal movement
Source: Mov Ecol. 2017 Jan 26;5:1. doi: 10.1186/s40462-016-0093-6 (PMC5270370; doi:10.1186/s40462-016-0093-6)
Supplement: Additional file 1 — Log-Normal Von Mises HMMs. (PDF 231 kb) [file 40462_2016_93_MOESM1_ESM.pdf]

Log-Normal Von Mises HMMs

In the main text of this manuscript, we showed results for Cat 1 using HMMs that ignored turning angles. Here, we show results from models that incorporate turning angles. We analyze ΔBIC (Figure S1) and step-length (Figure S2, S3) diagnostics. Our conclusions from the main text persist across all cats; namely, (1) temporally heterogeneous models select fewer BIC-optimal states and overall, lower BICs; (2) heterogeneous models capture the diurnal pattern and autocorrelations as described in the main text (except for cat 14, 15). Lastly, we plotted the densities of the corresponding step-length and turning angle distributions given the movement states. The correlation between step-length and turning angles are consistent with the animal movement literature (i.e. movement states with longer step-length also have a narrower range of turning angles; those with short and intermediate steps have a wider range of turning angles). but short and intermediate steps are hard to interpret biologically (Figure S4-S7).

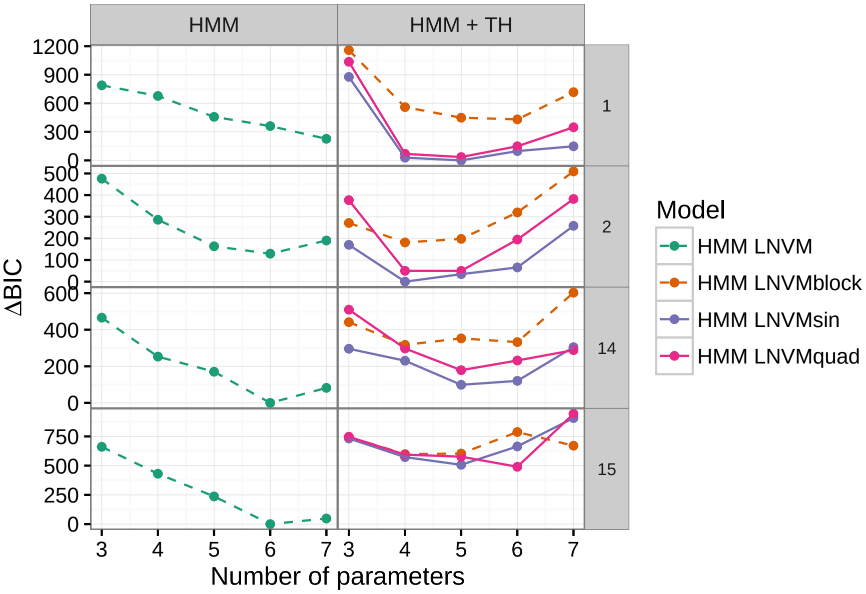

Figure S1: Relative BIC values for HMMs with turning angles (log Normal-von Mises models), for all cats: see Figure 3 in main text for details.

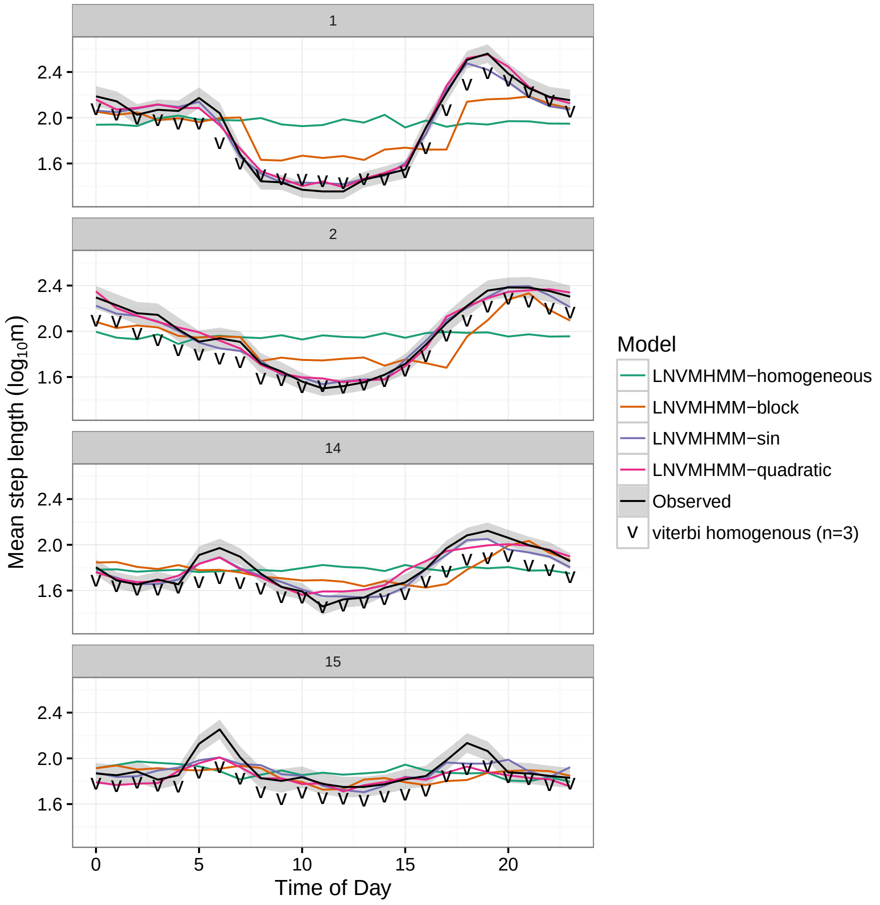

Figure S2: Out-of-sample predicted and observed mean step length by time of day for all log Normal-von Mises models, for all cats: see Figure 4 in main text for details.

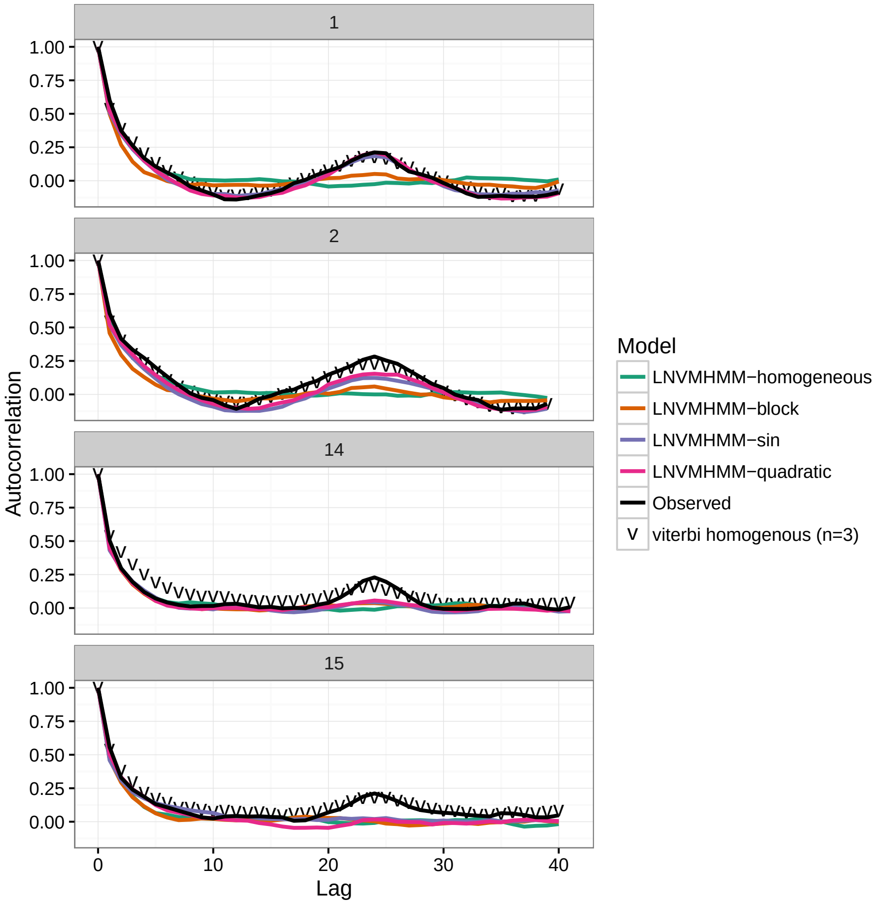

Figure S3: Out-of-sample predicted and observed autocorrelation functions for step length for all log Normal-von Mises models, for all cats: see Figure 5 in main text for details.

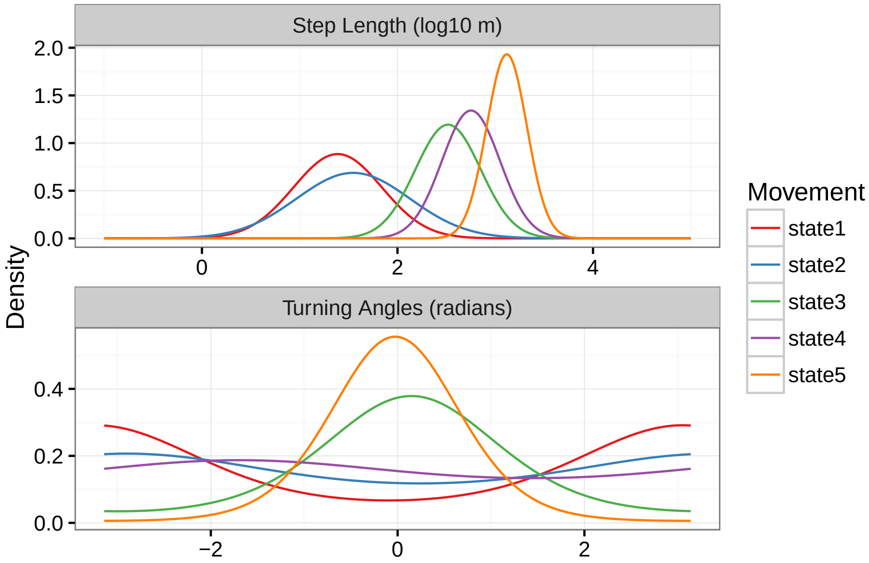

Figure S4: Predicted distributions of step length and turning angle by movement state, cat 1. States with long expected step-lengths (4 and 5) have relatively narrow turning angle distributions centered at 0∘. Those with short and intermediate step-lengths (1-3) have wider turning angle distributions. State 1, the shortest step-length state, shows a bimodal turning angle distribution probably driven by GPS error [1]. x-axis units are $\log_{10}$m (step length) and radians (turning angles).

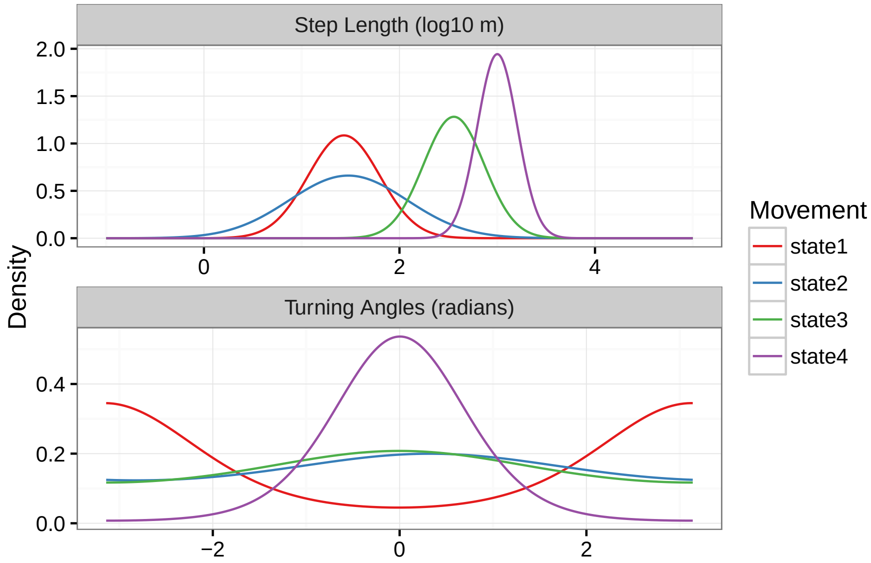

Figure S5: Predicted distributions of step length and turning angle by movement state, cat 2. See Figure S9 for details.

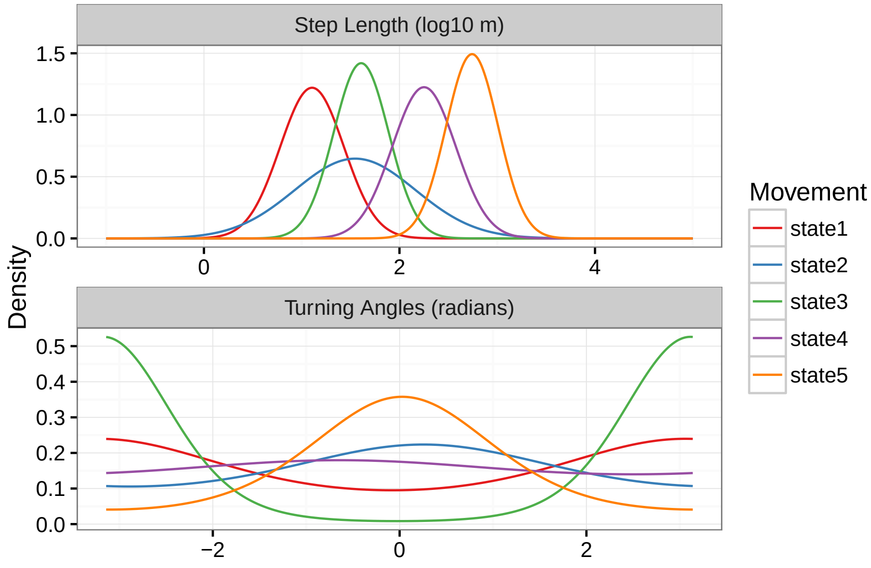

Figure S6: Predicted distributions of step length and turning angle by movement state, cat 14. See Figure S9 for details.

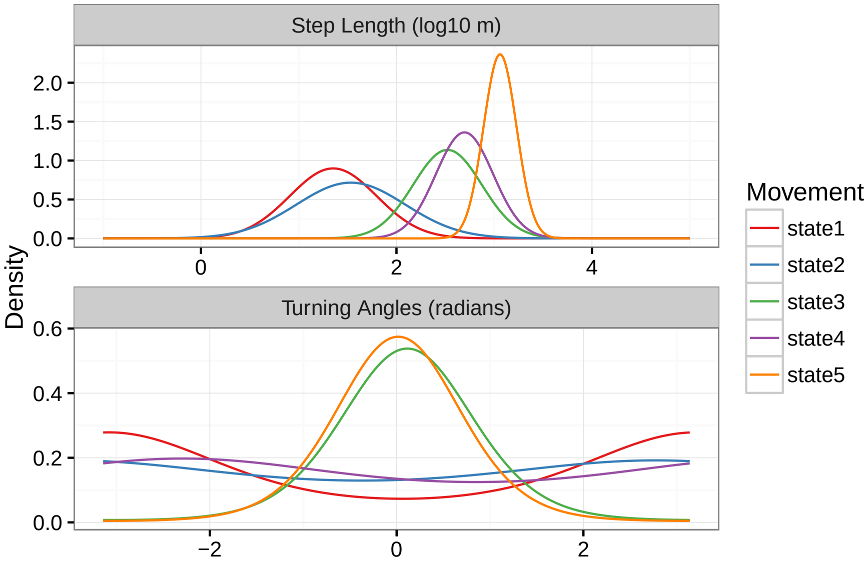

Figure S7: Predicted distributions of step length and turning angle by movement state, cat 15. See Figure S4 for details.

References

[1] Hurford, A.: GPS Measurement Error Gives Rise to Spurious 180° Turning Angles and Strong Directional Biases in Animal Movement Data. PLOS ONE **4**(5), 5632 (2009). doi:10.1371/journal.pone.0005632
