# Supplementary material for: Incorporating periodic variability in hidden Markov models for animal movement
Source: Mov Ecol. 2017 Jan 26;5:1. doi: 10.1186/s40462-016-0093-6 (PMC5270370; doi:10.1186/s40462-016-0093-6)
Supplement: Additional file 2 — Simulation experiment. (PDF 58.8 kb) [file 40462_2016_93_MOESM2_ESM.pdf]

Simulation Experiment

To check the effect of GPS error on estimation of the number of states, we simulated 100 step-length sequences from a two-state HMM with time-heterogeneous (sinusoidal) transition probability (Eq. 5 in the main text) with random transition parameters, using the simulation method described in the main text. The step-length and turning angle distributions for state 1 and 2 are:

$$SL \mid s_1 \sim \text{log-Normal}(\mu = 0, \sigma = 1) \quad (1)$$
$$TA \mid s_1 \sim \text{von-Mises}(\alpha = 0, \kappa = 0) \quad (2)$$
$$SL \mid s_2 \sim \text{log-Normal}(\mu = 2, \sigma = 1) \quad (3)$$
$$TA \mid s_2 \sim \text{von-Mises}(\alpha = 0, \kappa = 10) \quad (4)$$

where μ is the mean and σ is the standard deviation on the log scale; α is the mean direction; and κ is the concentration. Figure S8 illustrates the frequencies with which different BIC-optimal states are estimated for 100 simulated realizations, fitted using either homogeneous or heterogeneous transition probabilities.

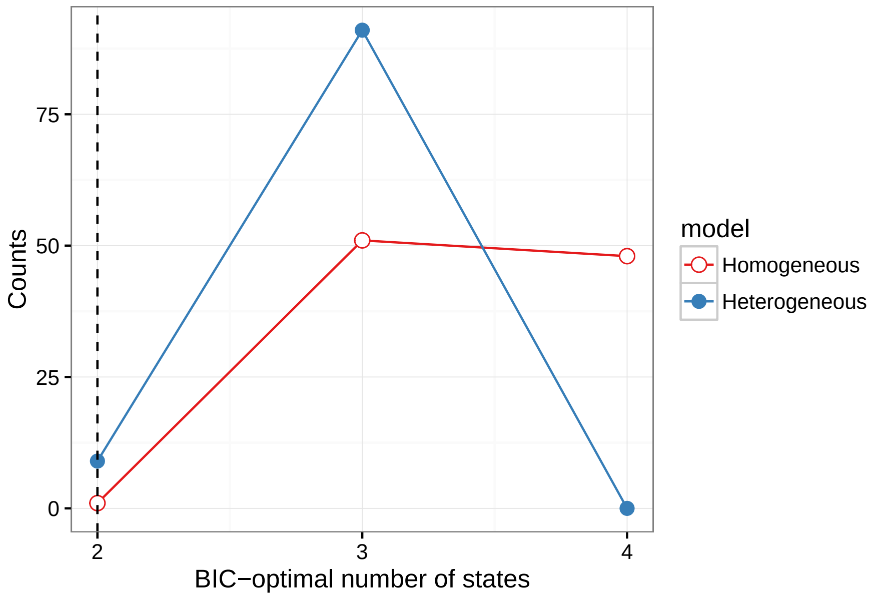

Figure S8: Simulation with GPS error. For details, refer to Figure 1 in main text (bottom-right panel).
