# Supplementary material for: Incorporating periodic variability in hidden Markov models for animal movement
Source: Mov Ecol. 2017 Jan 26;5:1. doi: 10.1186/s40462-016-0093-6 (PMC5270370; doi:10.1186/s40462-016-0093-6)
Supplement: Additional file 3 — HMM Analysis for Cat 2, 14, 15. (PDF 90 kb) [file 40462_2016_93_MOESM3_ESM.pdf]

Cat 2, 14, 15

In the main text of this manuscript, we showed results for Cat 1 only. Here, we present plots showing the comparison of BIC-optimal state predictions (Figure S9), adjusted BIC comparisons (Figure S10), average step-length by time of day from out-of-sample predictions (Figure S11) and autocorrelation from out-of-sample predictions (Figure S12) for all four cats (1, 2, 14, and 15). Most conclusions are similar across all cats; in a few cases the BIC-optimal number of states is lower, and the BIC advantage of heterogeneous over homogeneous models is smaller or reversed, for the cats with the smallest data sets (14 and 15).

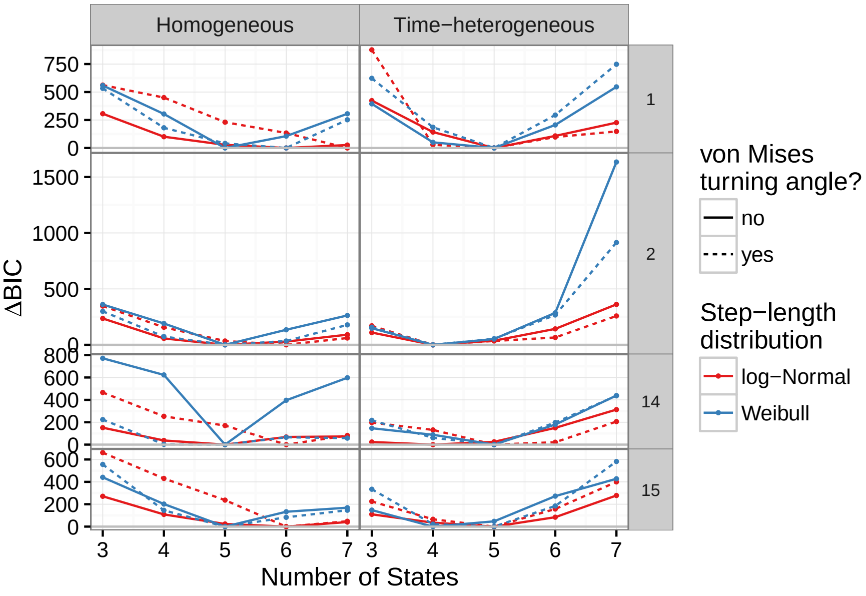

Figure S9: Relative goodness of fit for homogeneous models with different step-length distributions and with and without turning angles: see Figure 2 in main text for details.

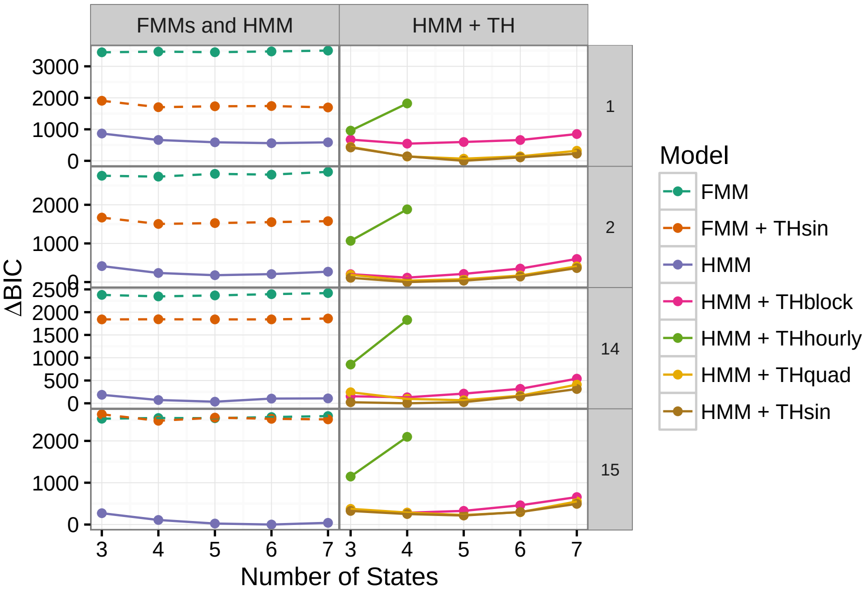

Figure S10: Relative BIC of homogeneous FMMs and HMMs with differing number of movement states: see Figure 3 in main text for details.

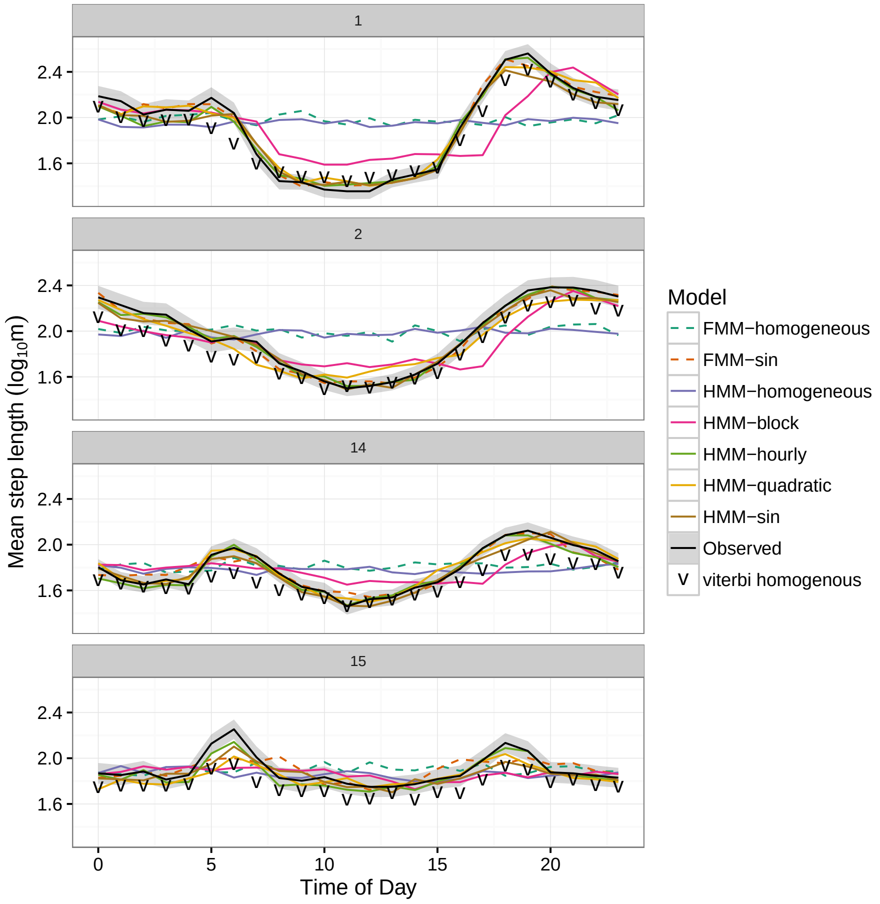

Figure S11: Out-of-sample predicted and observed mean step length by time of day for all models: see Figure 4 in main text for details.

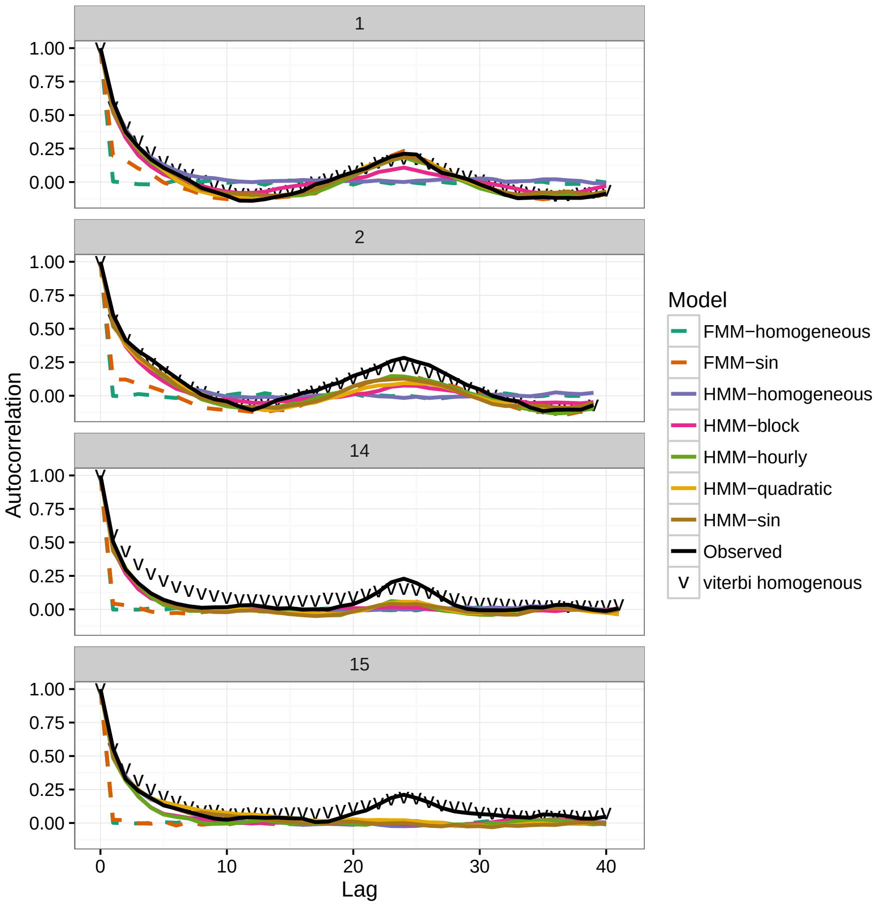

Figure S12: Out-of-sample predicted and observed autocorrelation functions for all models: see Figure 5 in main text for details.
